# High-density linkage map construction and mapping of seed trait QTLs in chickpea (*Cicer arietinum* L.) using Genotyping-by-Sequencing (GBS)

**DOI:** 10.1038/srep17512

**Published:** 2015-12-03

**Authors:** Subodh Verma, Shefali Gupta, Nitesh Bandhiwal, Tapan Kumar, Chellapilla Bharadwaj, Sabhyata Bhatia

**Affiliations:** 1 National Institute Of Plant Genome Research, Aruna Asaf Ali Marg, PO Box No. 10531, New Delhi, 110067, India; 2 Indian Agricultural Research Institute, Pusa Campus, New Delhi, 110012, India

## Abstract

This study reports the use of Genotyping-by-Sequencing (GBS) for large-scale SNP discovery and simultaneous genotyping of recombinant inbred lines (RILs) of an intra-specific mapping population of chickpea contrasting for seed traits. A total of 119,672 raw SNPs were discovered, which after stringent filtering revealed 3,977 high quality SNPs of which 39.5% were present in genic regions. Comparative analysis using physically mapped marker loci revealed a higher degree of synteny with *Medicago* in comparison to soybean. The SNP genotyping data was utilized to construct one of the most saturated intra-specific genetic linkage maps of chickpea having 3,363 mapped positions including 3,228 SNPs on 8 linkage groups spanning 1006.98 cM at an average inter marker distance of 0.33 cM. The map was utilized to identify 20 quantitative trait loci (QTLs) associated with seed traits accounting for phenotypic variations ranging from 9.97% to 29.71%. Analysis of the genomic sequence corresponding to five robust QTLs led to the identification of 684 putative candidate genes whose expression profiling revealed that 101 genes exhibited seed specific expression. The integrated approach utilizing the identified QTLs along with the available genome and transcriptome could serve as a platform for candidate gene identification for molecular breeding of chickpea.

Chickpea (*Cicer arietinum* L.) is a self pollinated, diploid (2n = 2x = 16) annual grain legume crop with a genome of size ~738 Mb^1^. It is mostly cultivated in arid and semi-arid areas and ranks third in production among food legumes worldwide^1^,^2^. Being a rich and economic source of proteins, vitamins and minerals, chickpea seeds are an important commodity, especially in the diet of the poor and vegetarian population of developing countries^3^. The economic importance of chickpea seed therefore necessitates genetic and genomics studies especially related to traits such as seed yield as this is a major target of chickpea breeding. Attempts have been made to map QTLs responsible for seed traits in chickpea^3^,^4^,^5^. However, since high density maps were not used for such purposes, it resulted in QTLs with low resolution. Moreover, due to the unavailability of the chickpea genome sequence earlier, identification of candidate genes for yield related traits had also not been possible.

A linkage map, densely populated with molecular markers, is a prerequisite for successful QTL identification^6^. Amongst all types of molecular markers, single nucleotide polymorphisms (SNPs) have proven to be the marker of choice because of their ubiquitous presence in high numbers, uniform distribution, biallelic nature and high heritability^7^,^8^. Moreover, variations at a single nucleotide level within genes may sometimes lead to changes in gene function that may be majorly responsible for phenotypic differences^9^. Therefore, discovery of SNPs linked to important agronomical traits is of utmost importance.

Recent developments in next generation sequencing (NGS) technologies have expedited the discovery of molecular markers, especially SNPs at the genome-wide level in a cost-effective manner^10^. Coupled with this has been the development of diverse methods for SNP genotyping, such as Illumina’s BeadArray™ technology based GoldenGate and Infinium® assays, Life Technologies TaqMan assay and KBiosciences Competitive Allele Specific PCR (KASPar™)^11^. In addition to these, new genotyping methods have been developed that utilize restriction enzymes for genome complexity reduction in combination with NGS for SNP discovery and genotyping such as the restriction site associated DNA sequencing (RAD-seq) and Genotyping-by-Sequencing (GBS)^12^,^13^. Among these, the GBS approach has proven to be the method of choice which allows sequencing, discovery and genotyping of thousands of SNPs in a single step^12^,^14^. GBS involves a relatively simple and short library preparation protocol that requires small amount of starting DNA (100–200 ng) and no random shearing and size selection steps^15^. Being an efficient and cost-effective approach, it has been widely used in various species for diverse genetic applications such as in apple^16^, barley^17^,^18^, maize^19^,^20^, rice^21^, wheat^18^,^22^, and soybean^15^. However, in a crop legume such as chickpea, which has a narrow genetic base, GBS needs to be exploited for high density linkage map construction and powerful QTL mapping of important traits.

With this in view, the following objectives were undertaken in this study: (i) GBS based identification of SNPs in an intra-specific mapping population (*C. arietinum* SBD377 X BGD112) contrasting in seed traits, (ii) utilizing the genomic positions of discovered SNPs for comparative analysis between *desi* and *kabuli* chickpea as well as between chickpea and *Medicago* and soybean genomes, (iii) generating a high resolution intra-specific linkage map of chickpea, (iv) identifying QTLs related to seed traits viz. seed weight, seed number, seeds per pod and pods per plant, and (v) predicting putative candidate genes underlying mapped QTLs that may be involved in genetic regulation of seed related traits in chickpea.

## Results

### Genome wide identification of SNPs using GBS approach

**Figure 1:**
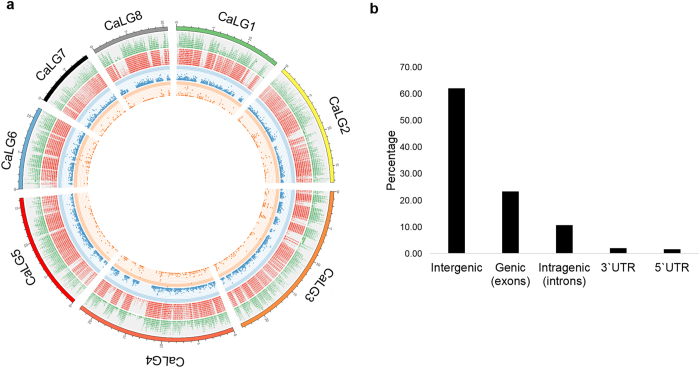
Distribution and structural annotation of SNPs. (**a**) Distribution of SNPs detected on each chickpea chromosome (5 kb window size) are shown in the Circos diagram. Track 1 represents the 8 chickpea chromosomes (CaLG1-8) in different colours. Tracks 2, 3, 4 and 5 represent genes, exons, raw SNPs and filtered SNPs, respectively. Different panels are represented by different colors as indicated. (**b**) Distribution of SNPs on the basis of their location in different genomic regions.

For genome-wide detection of SNPs from chickpea using GBS approach, the 5 base cutter restriction enzyme ApeKI was used to digest genomic DNA and construct the 96-plex GBS libraries of the RILs and the parents of the intra-specific mapping population (*C. arietinum* SBD377 X BGD112). Sequencing was carried out in a flow cell lane of an Illumina HiSeq 2000 sequencer and 209.34 million reads (100 bp length) were generated. The sequencing data obtained in our study has been deposited in the NCBI-short read archive (SRA) database^23^ under the accession number SRX1022562. Quality Control (QC) was carried out by applying various filtering criteria such as removal of barcode and ApeKI overhang sequences and maintaining a phred quality score ≥15 for at least 80% of the nucleotides. Finally 171.14 million (81.75%) high quality filtered reads successfully passed the QC steps as the remaining reads were filtered out due to the lack of proper layout of barcodes and restriction sites. The average number of reads among individuals was 1,801,433 (Fig. S1a). The variation in the number of reads between individuals was 29.88%. Further, the TASSEL-GBS analysis pipeline^24^ was used for SNP mining from the sequence data and 119,672 raw SNPs were identified. The average call rate per individual was 96.51% (Fig. S1b). The SNP data was subsequently filtered for identifying putative markers using the criteria of 65% missing values across the genotyped individual and MAF ≥0.15. A total of 3,977 SNPs were identified after imputation and were designated as “CaGSNP”. The list of SNPs along with their flanking sequences has been provided in Supplementary Table S1. The SNPs were found to be distributed across all 8 chickpea chromosomes as illustrated in Fig. 1a, Supplementary Table S2. The number of raw and filtered SNPs and their frequency (number per 100 kb) varied across chromosomes and closely mirrored the distribution of genes and exons. The largest number of raw SNPs was observed on chromosome 3 (12,047). The frequency of raw SNP per 100 Kb was highest on chromosome 8 (56.69) and lowest on chromosome 2 (37.26). However, maximum number of SNPs, after filtering, was identified on chromosome 4 (179). The SNPs detected were also classified as transitions or transversions. The occurrence of both types of transitions- C/T (26.05%) and A/G (22.63%) was higher than any of the transversions.

### Gene annotation

The chickpea genome annotation project database^25^ was used to delineate the location of the GBS derived 3,977 SNPs in the genomic regions: intergenic, genic (exons), intragenic (introns) and UTRs. The maximum (62.08%) were located in intergenic regions (Fig. 1b, Table S3). Of the remaining, the largest numbers of SNPs were found to be located within exons (23.37%) followed by introns (10.76%), 3′ UTR (2.09%) and 5′ UTR (1.71%). Altogether, 866 gene models were observed which contained SNPs in their exons (Table S3). Several genes with two or more SNPs were also identified. The genes Ca_04685.1 and Ca_06825.1 encoding “Polyadenylate binding protein-3-like” and “Subtilisin like protease SDD1” respectively, had the highest number of SNPs in their exons.

To analyze the functional relevance of SNPs, GO (gene ontology) enrichment analysis was performed for the genes carrying SNPs in their coding regions using in-house custom perl scripts. Of the 866 genes, 694 had a significant match with entries in nr database of NCBI. These genes were classified into the three major GO categories i.e. biological process, molecular function and cellular component (Fig. S2). Under the “biological processes” category, most of the genes were associated with ‘metabolic processes’ followed by ‘cellular processes’. Most of the “molecular functions” category included ‘catalytic activity’ followed by ‘binding activity’. In the “cellular component” category, the most represented components were ‘cell’ followed by ‘membrane’.

Transcription factors (TFs) are the key players in mediating transcriptional regulation and hence SNP containing TF genes were identified and categorized into TF families. HMM (Hidden Markov Model) profiles of transcription factors were built using HMMBUILD (HMMER 3.0)^26^ and used for classifying SNP containing genes into TF families. Out of 866 genes, 90 genes were identified to be TFs which belonged to 35 known TF families. The predominant families were bHLH (15), CAMTA (10) and AP2 (8) followed by other families such as ERF, ZF-HD and B3 transcription factor families (Fig. S3).

### Comparative analysis between chickpea and other legumes

Flanking sequences (50 bp on either side) of the 3,977 SNPs were extracted and aligned to the genome assemblies of *kabuli* chickpea^1^ and *desi* chickpea^2^, using BLASTN (version 2.2.25+). Out of 3,977 maker loci, chromosomal positions of 3,147 (79.13%) SNPs could be obtained from the *kabuli* chickpea genome whereas only 1,448 (36.41%) markers could be mapped on the *desi* chickpea chromosomes. Hence the physical locations of 3,147 SNP markers on the 8 chromosomes of *kabuli* chickpea genome (designated Ca1-Ca8) were utilized for synteny analysis with other legumes namely soybean and *M. truncatula*. Comparison of chickpea with *Medicago* revealed 684 unique chickpea loci that showed significant similarities with 970 loci on the *Medicago* chromosomes (Fig. S4a, Table S4). A number of chickpea loci showed multiple (>2) matches with *M. truncatula*. The highest number of 138 loci from Ca4 of chickpea showed maximum similarity to 136 loci on MtChr1. Similarly, loci from Ca1 and Ca6 exhibited high similarity with 90 loci each of MtChr2 and MtChr4 respectively. Similarly, Ca3 shared common marker loci with 71 loci on MtChr7 followed by 52 between Ca8 and MtChr5. The minimum numbers of matches were found between Ca7 (56) and MtChr4 (28) (Table S4).

Comparison of chickpea and soybean sequences revealed that 580 chickpea loci had significant matches with 1,200 loci on the soybean chromosomes (Fig. S4b, Table S5) wherein, each of the chickpea loci showed hits with two or more genomic regions of soybean. Here also, the highest numbers of 131 SNP loci from Ca4 and 121 SNP loci from Ca6 found matches with 67 loci on Gm10 and 60 loci on Gm08 respectively. Similarly, 86 loci from Ca3 showed maximum number of matches with 46 loci on Gm19 and 41 on Gm03 (Table S5). The least number of matches were observed between Ca7 and Gm03 (13) and Gm17 (13) followed by Ca8 and Gm01 (10). In brief, a significant number of the chickpea SNP loci co-localized with more than two loci on different chromosomes of *Medicago* and soybean reflecting the occurrence of duplication events in the respective genomes.

**Figure 2:**
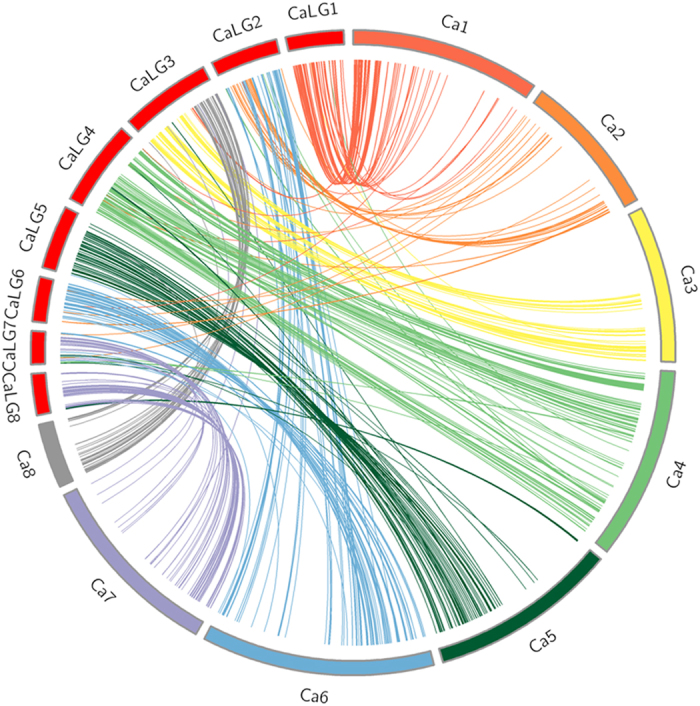
Comparison of *desi* and *kabuli* chickpea genomes using GBS derived SNP markers. Comparative analysis between both the genomes is visualized using Circos plot. *Desi* chromosomes are indicated as ‘CaLG’ and *kabuli* chromosomes are represented as ‘Ca’.

Comparison of 3,147 SNP loci between *desi* and *kabuli* chickpea genomes^1^,^2^ revealed 1,364 common SNP loci with significant homology (Fig. 2, Table S6). All *kabuli* chromosomes were found to be highly syntenic with their corresponding *desi* chromosomes such as *kabuli* Ca3 which showed maximum synteny (99.48%) with *desi* CaLG3, followed by *kabuli* Ca1 with *desi* CaLG1 (91.33%), *kabuli* Ca4 with *desi* CaLG4 (87.79%), *kabuli* Ca5 with *desi* CaLG5 (66.43%), *kabuli* Ca6 with *desi* CaLG6 (55.72%) and *kabuli* Ca2 with *desi* CaLG2 (53.06%). However, the *kabuli* chromosomes Ca7 and Ca8 showed higher synteny with the non-corresponding *desi* CaLG8 and CaLG3, respectively.

### Construction of a high resolution intra-specific linkage map

**Figure 3:**
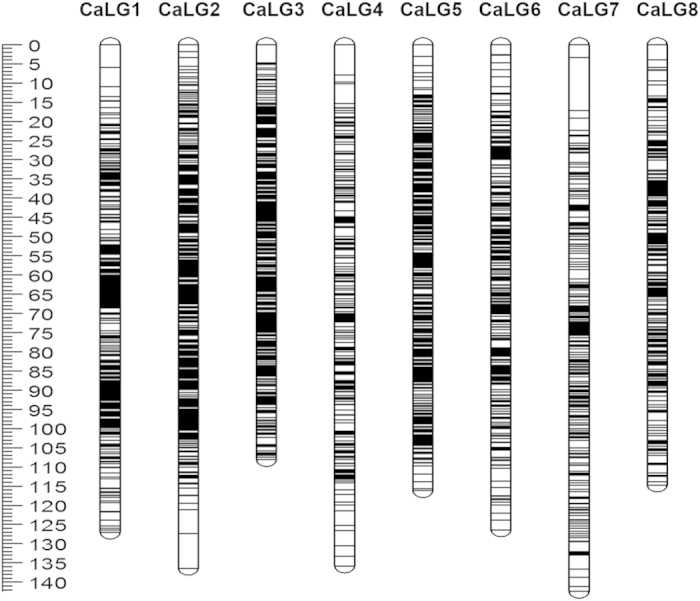
Intra-specific genetic linkage map of chickpea constructed using the RIL population derived from the parental lines SBD377 X BGD112. The scale shown on the left is in cM.

The GBS data of the RILs arising from an intra-specific cross of chickpea *C. arietinum* (SBD377 X BGD112) was utilized for generating the intra-specific linkage map. The genotyping data of 3,977 SNP loci along with the data of 135 previously developed EST-based markers and genomic SSR markers^27^ were used for mapping. A linkage map was generated that comprised of 3,363 markers on 8 linkage groups which included 3,228 SNP markers generated in this study and the 135 earlier developed markers (Fig. 3, Table S7), thereby indicating that 81.16% (3,228 out of 3,977) of the total SNPs could be successfully assigned genetic positions. LGs were designated as CaLG1-CaLG8 and were numbered based on the presence of common markers with reference to the earlier published maps^27^,^28^,^29^,^30^. The total length of the linkage map was 1006.98 cM with LG7 (142.43 cM) being the largest and LG3 (108.12 cM) being the smallest. The number of markers per linkage group varied from 254 (LG4) to 584 (LG2), with an average of 420.37 markers per linkage group. The average marker density was 0.33 cM with LG3 being most dense (0.19 cM) and LG7 being the least (0.54 cM). Few (only 16) gaps were observed, most of which were less than 3 cM in length with the largest being 13.85 cM on LG7. A summary of the constructed genetic map is presented in Table 1.

### Evaluation of phenotypic data

Phenotyping data was collected for number of seeds per pod (SP), number of pods per plant (PP), 100-seed weight (SW) and number of seeds per plant (SN) over two consecutive years, 2012 and 2013, with two replications each for the F_11_ mapping population (*C. arietinum* SBD377 X *C. arietinum* BGD112). Significant differences for four traits were observed within the mapping population in F_11_ generation and parental genotypes. Descriptive statistics of the trait analysed in this study have been summarized in Table 2. Normal frequency distribution was observed for all the traits analysed except for PP which was slightly deviated from normal distribution (Fig. S5). Pearson`s correlation test between the phenotypic traits showed that SW correlated significantly with all the other traits except PP, while SN correlated with all the traits except SP with negative correlation with SW. SP correlated negatively with SW. PP was positively correlated with SN. The highest degree of correlation was observed between SN and PP (r = 0.757). Phenotypic correlations between the analysed traits have been presented in Table 3.

### Identification of QTLs

**Figure 4:**
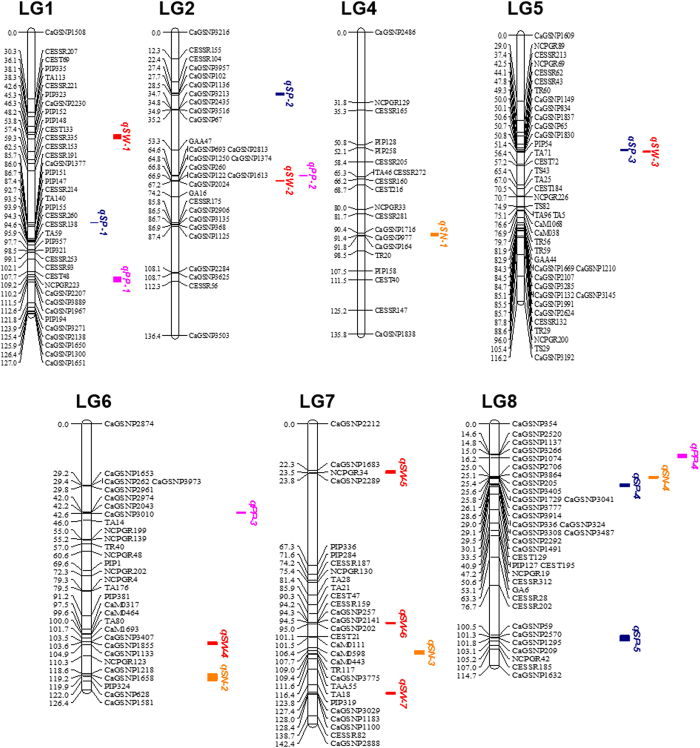
Location of QTLs on the genetic linkage map of chickpea developed from the cross SBD377 X BGD112. Distances among markers are indicated in cM to the left of the linkage groups; names of markers are shown on the right. Only those SNP markers are shown which were in and around the QTL regions. QTLs are depicted as colored vertical bars to the right of the linkage groups. LG3 is not shown because no QTL was detected on it.

For identification of QTLs, genotyping data of the 3,363 markers mapped on the intra-specific linkage map was integrated with the phenotyping data of the 4 traits described above and analysed using composite interval mapping (CIM) method. A total of twenty QTLs were identified for the following traits- SW, SN, SP and PP which were distributed on all the LGs except LG3 (Fig. 4). The LOD thresholds ranged from 3.01 (*qSN-2*, *qSP-3*) to 5.52 (*qSW-3*). The identified QTLs have been summarized in Table 4.

#### Seed weight

Seven QTLs were detected for seed weight which mapped on LGs 1, 2, 5, 6 and 7 with the LOD thresholds ranging between 3.02 (*qSW-1*) to 5.52 (*qSW-3*) (Fig. 4). At all the loci except *qSW-6*, the alleles from parent *C. arietinum* BGD112 favoured seed weight. The QTLs explained phenotypic variance in the range of 10.07% (*qSW-7*) to 22.31% (*qSW-5*).

#### Seed number per plant

Four QTLs for seed number per plant were detected on LGs 4, 6, 7 and 8 with LOD thresholds of 3.01 to 4.76. The phenotypic variance explained by these QTLs ranged between 9.97% (*qSN-2*) and 18.84% (*qSN-1*). Phenotypic effects contributed from *C. arietinum* SBD377 were observed for *qSN-3* and *qSN-4* showing negative additive effects, while phenotypic effects derived from *C. arietinum* BGD112 were observed for *qSN-1* and *qSN-2* showing positive additive effects.

#### Number of seeds per pod

Five QTLs pertaining to the trait number of seeds per pod could be mapped on LGs 1, 2, 5 and 8. The LOD thresholds varied from 3.01 (*qSP-3*) to 4.98 (*qSP-1*). Phenotypic variance varied from 10.84% (*qSP-5*) to 17.91% (*qSP-1*).

#### Number of pods per plant

Four QTLs were detected for number of pods per plant on LGs 1, 2, 6 and 8, respectively with additive effects ranging from 49.54 to 16.14 and % variance from 29.71 to 12.81. The parent BGD112 contributed the favorable allele at all loci except *qPP-4*.

### Identification of candidate genes

**Figure 5:**
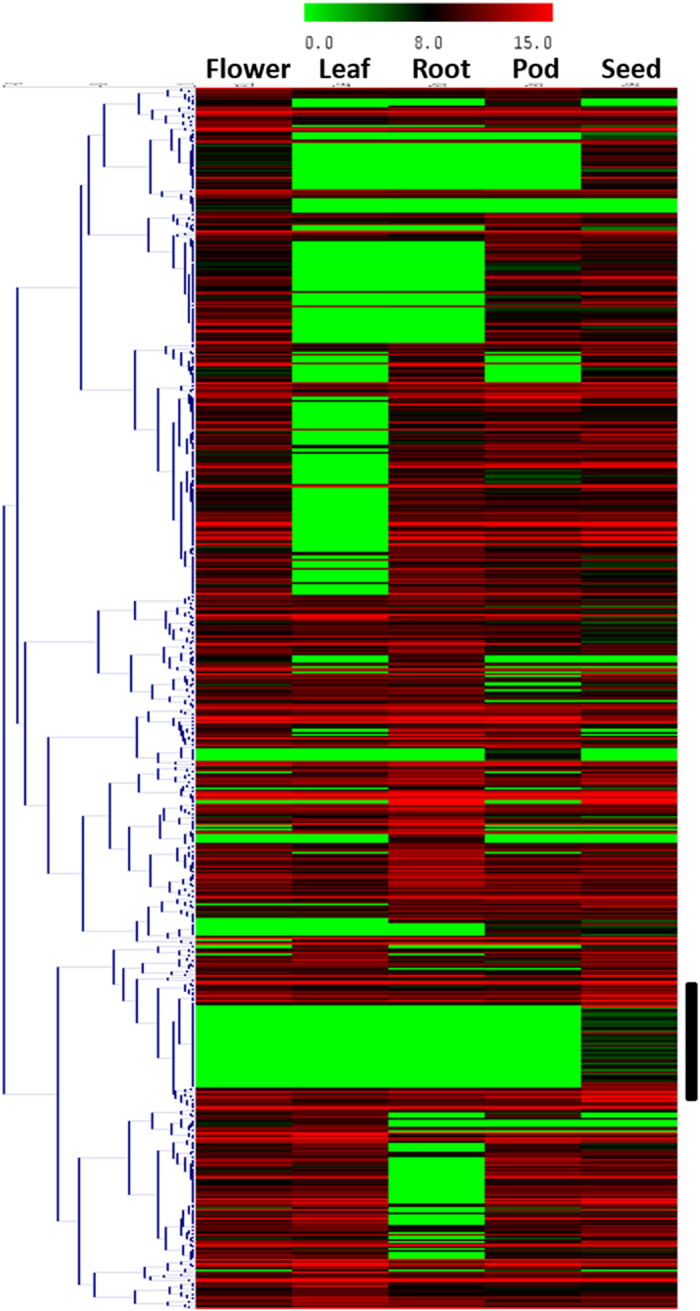
Differential expression patterns of identified putative candidate genes in various chickpea tissues. Block represents genes having higher expression in seed tissue.

Due to the availability of the whole genome sequence of chickpea it became possible to identify potential candidate genes underlying QTLs mapped in this study. In order to do this, only those QTLs with significantly high LOD scores and phenotypic variance were selected. Further, these QTLs were assessed for the location of their underlying markers in the whole genome. Only those QTLs that had interval markers on the same chromosomes were selected. Hence in all, 5 QTLs including 2 QTLs for seed weight (*qSW-3* and *qSW-4*), 2 for seeds per pod (*qSP-1* and *qSP-4*) and 1 for pods per plant (*qPP-2*) were selected for candidate gene analysis. Next, the sequences of the proximal markers underlying the QTLs were used to extract the corresponding genomic regions from the draft genome sequence of the *kabuli* chickpea^1^. Corresponding to these 5 QTLs, 9.72 Mb region spanning the QTLs was extracted from the available *kabuli* genome sequence of chickpea (Table S8). This region was subjected to structural and functional annotation and a total of 684 candidate genes could be identified that have been listed in Supplementary Table S9. Further, in order to identify the set of most robust candidate genes, all the 684 genes were analysed for their *in silico* expression profiles using the available transcriptome data of various chickpea tissues such as leaf, root, flower-bud, pod and seed^31^,^32^. Among the 14 genes of *qSW-3* and 604 genes of *qSW*-4, 3 and 84 genes respectively showed preferential expression in seed tissues in comparison with other tissues (Fig. 5, Table S9). These set of candidate genes consisted of protein encoding tubby like F-box protein 5, pentatricopeptide repeat containing protein, LOB domain containing protein, diacylglycerol o-acyltransferase 2-like protein, seed linoleate 9 s-lipoxygenase-3-like protein and c3hc4-type ring zinc finger protein. Similarly, among the genes of *qSP-1* and *qSP-4*, 9 and 4 genes were found to have seed specific expression. In case of *qPP-2*, out of 10 genes, only 1 gene was highly expressed in seed in comparison to any other tissue. Hence from the 5 QTLs, 101 genes were predicted to be the putative candidates associated with seed traits.

## Discussion

This study clearly demonstrated that mapping of QTLs to identify genetic regions and candidate genes underlying agronomically important traits could be efficiently done using the Genotyping by Sequencing (GBS) approach. This utilizes sequencing of a restriction enzyme-targeted fraction of the genome using next-generation sequencing technology^14^. GBS does not require complete genome sequencing of all individuals and parents of a mapping population, only a targeted sequencing approach is necessary. Due to its high throughput efficiency, GBS has recently been used for SNP identification and mapping in several plant species including chickpea^18^,^33^,^34^,^35^,^36^,^37^. In chickpea, most of the genetic linkage maps constructed so far have employed molecular markers such as genomic and genic SSRs^29^,^38^,^39^, DArT markers^40^,^41^ and SNPs^30^. However, very recently GBS has been used for linkage map construction and QTL identification in chickpea^36^,^37^. In the present study, we utilized the GBS platform to sequence an intra-specific mapping population of chickpea contrasting for seed traits. This RIL population provided a new source of genetic variation which could be used to identify loci associated with seed traits. Such high-throughput analysis of diverse germplasm in chickpea will accelerate the process of crop improvement especially when the *desi* and *kabuli* chickpea draft genome sequences have become available^1^,^2^. In this study, 119,672 raw SNPs were discovered from the genome directly using the TASSEL pipeline^24^. However, SNPs were found to be clustered in several places on chromosomes (Fig. 1a). One of the reasons for this could be the use of specific restriction enzymes used for GBS library preparation which generate fragments only from targeted regions of the genome^15^ necessary for complexity reduction during sequencing. After filtering, based on missing data in genotypes and minor allele frequency considerations, 3,977 SNPs were found appropriate for use in linkage mapping. An important advantage of GBS approach is that a reference genome is not necessary for SNP calling and genotyping. However, availability of a reference genome bestows additional benefits as it allows proper alignment and ordering of the sequenced tags and helps in imputing low coverage data^13^. Since both parents of the mapping population were of *desi* type, the publically available genome sequence of the *desi* cultivar, ICC4958^2^ was utilized for assembly and alignment of sequence tags. Missing data is one of the major disadvantages of GBS^42^ and arises due to technical limitations of GBS technology such as unequal sequencing of reads across the SNP loci and low coverage of sequence^43^. Problems of missing data can be dealt with by performing imputation. There are several imputation algorithms, most of which are based on haplotype construction and use consensus haplotypes to impute missing markers^19^. In the present study, haplotype-based algorithm of Beagle^44^ was utilized to overcome the issues related to missing data and to improve the SNP information.

Comparative mapping between genomes can reveal the genetic basis of conservation and differences among related species^45^. Several previous studies have reported syntenic relationships between chickpea and other legumes^30^,^46^. However, most of these studies were based on limited number of marker loci and utilized genetic maps for comparisons. In this study, we took advantage of the available whole genome sequences of legumes and performed comparative analysis using SNP loci that were physically assigned to the genomes. Approximately 79.13% (3,147/3,977) of the identified SNPs could be successfully aligned to the *kabuli* chickpea chromosomes whereas only 36.41% could be mapped on *desi* chromosomes. This was because the *kabuli* genome was a better anchored genome as compared to *desi* which was more fragmented into scaffolds. Hence, SNPs that were located on the *kabuli* chromosomes were used to investigate the synteny between chickpea and *Medicago* as well as soybean. A significantly higher number of unique marker loci of chickpea showed orthology with *Medicago* in comparison to soybean, suggesting that chickpea is closer to *Medicago* than to soybean in agreement with earlier reports^1^,^30^,^46^. Moreover, though intra and inter chromosomal duplication blocks are known to be present in chickpea, recent whole genome duplication (as observed in soybean) have been reported to be absent in chickpea^2^. The same was observed in present study (Fig. 2 and Fig. S4). The information from the comparative genome analysis will be highly valuable to understand the evolution of chickpea with respect to the other legumes.

Further, we also performed comparative analysis using physically mapped GBS markers between *desi*^2^ and *kabuli*^1^ chromosomes. Large regions of similarity between the corresponding chromosomes were observed. However, significant level of similarity was also identified between unrelated chromosomes which included *kabuli* chromosomes Ca7 and Ca8 showing large number of matches with *desi* chromosomes CaLG8 and CaLG3 respectively. These structural differences between the *desi* and *kabuli* genomes were also observed by Ruperao *et al.*^47^ and were probably due the two genomes using different genetic maps to anchor their assemblies. Moreover in the available *desi* draft assembly^2^ only 124 Mb of the genome had been anchored to the LGs.

Chickpea, as most other legumes, is known to have a narrow genetic base^29^,^48^. Hence detection of polymorphism using the available marker technologies such as SSRs and SNPs has been limited. The levels of inter-specific polymorphism detected using SSR markers varied from 16%^38^ to 36%^29^ across related species whereas intra-specific polymorphism has been as low as 9.5%^49^ within species. Similarly, variation detection based on SNPs has also shown similar trends^30^,^48^. Narrow genetic base coupled with low levels of intra-specific polymorphism necessitated that the high resolution genetic linkage maps be constructed by exploiting the inter-specific polymorphisms between *C. arietinum* and its wild progenitor *C. reticulatum*^29^,^30^,^40^ whereas very few high density intra-specific linkage maps of chickpea are available. However, with the availability of robust technologies such as GBS, it became possible to accurately detect sequence variations at high resolution even within closely related *desi* genotypes (SBD377 X BGD112). The 3,977 most robust SNPs that were identified were used to construct one of the densest intra-specific maps of chickpea having 3,363 mapped locations at an average marker density 0.33 cM which is better than those reported earlier even for inter-specific maps (1,336 markers, 0.5 cM, Deokar *et al.*^50^, 1,063 markers, 1.7 cM, Gaur *et al.*^30^, and 1,328 markers, 0.59 cM, Hiremath *et al.*^46^. This clearly established that the map reported here was one of the most saturated intra-specific maps of chickpea.

The high density linkage map generated in this study was utilized for identifying QTLs related to seed yield which is a complex trait governed by a number of components such as seed weight, seed number per plant and seed number per pod. These components are therefore favorable targets for selection in breeding and are quantitatively inherited and controlled by a number of QTLs with individual genetic effects. The population used in the study showed a continuous distribution and variability in the traits analysed indicating their quantitative nature of inheritance. Negative correlations between SW and SP and SW and SN were observed indicating the possibility of yield component compensation, where an increase in one yield component leads to a decrease in another component due to competition for limited resources. Similar phenomenon was observed for yield components in common bean^51^ and also corroborated the results of Cho *et al.*^52^ in chickpea. The high density linkage map developed in this study enabled us to identify QTLs with more accuracy and high resolution. A total of 20 QTLs for 4 traits were detected which explained phenotypic variance ranging from 9.97% to 29.71%. These QTLs were present at unique loci with defined underlying markers on different LGs and none were shared by two or more traits. However some were present in close proximity to each other such as *qSP-3* and *qSW-3* on LG5 and *qSW-2* and *qPP-2* on LG2 (Fig. 4). Comparing our QTLs with the previous studies, a number of loci appeared to overlap or were present near the previously mapped QTLs. In particular, two QTLs, *qSW-2* and *qSW-3* were mapped in close proximity to the markers linked to QTLs for the same trait in different mapping populations in previous reports. For example, QTL for seed weight on LG2 (*qSW-2*) was located near GAA47 which has repeatedly been reported to be linked to the seed weight QTL^4^,^5^,^53^. Another QTL, *qSW-3*, was mapped on LG5. In the vicinity of *qSW-3* several other markers such as TS43^54^, TS82 and TA96^53^ and TR56^49^ were present which have been previously reported to be associated with the QTL region for seed weight. Hence in all likelihood, the genomic regions comprising of QTLs *qSW-2* and *qSW-3* were putatively the same that had been shown to be associated with seed weight in other populations^4^,^5^,^49^,^53^,^54^. However, the differences in the exact QTL positions may be due to the different populations, markers and analytical tools used in various studies as well as the resolution of the generated maps.

Another advantageous feature of this study was the integration of the map and QTL information generated here with the already available genome and transcriptome information of chickpea. Hence taking advantage of the available whole genome sequence of chickpea and the QTLs identified in this study it became possible to identify and narrow down our search for putative candidate genes which govern seed related traits. For this analysis, the *kabuli* genome sequence^1^ was used since it was better assembled than the more fragmented *desi* genome^2^ and there was a higher probability of locating the QTL interval markers on the same chromosomes. Genomic sequence underlying the QTLs was extracted from the available whole genome sequence of chickpea^1^ and putative genes were predicted from the targeted QTLs. Further, their seed specific expression profiles, as evidenced from the publicly available transcriptome data of several chickpea tissues^31^,^32^ were validated. Finally, 101 candidate genes were identified with several of them having predicted roles in seed development (Table S9). For instance, the gene encoding diacylglycerol o-acyltransferase was observed which is known to play a role in lipid synthesis and storage and has been reported to enhance seed oil content and seed weight in *Arabidopsis* when overexpressed^55^. Another gene encoding C3HC4 ring protein has been reported to be involved in plant growth and fruit development in *Nicotiana benthamiana*^56^. In addition, genes like pentatricopeptide repeat containing proteins, serine threonine protein kinase which have been shown to play prominent roles in seed development in different crops were also present amongst the putative candidate genes^57^,^58^,^59^. A gene encoding a sugar transporter also figured amongst the candidates and its role in accumulation of seed reserve and transport has been established in various crops including *Arabidopsis*^60^, wheat^61^ and faba bean^62^. In addition to these bHLH-18 like transcription factor and tubby like F-box protein showed significant expression in seed tissue thereby suggesting their association with seed functions. Apart from genes having preferential expression in seed, the QTL regions also harbored genes like ring-h2 finger protein, floral homeotic apetala-2 and transparent testa1 (TT1). RIE1, encoding a RING-H2 zinc-finger protein was identified that has been demonstrated to be essential for seed development in *Arabidopsis*^63^. Similarly, the role of AP2, whose role in regulation of seed size and weight has been demonstrated in *Arabidopsis*^64^. The transparent testa 1 (TT1) gene which has been shown to encode a seed specific regulatory factor involved in endothelium development and pigmentation in the seed coat^65^ was also present. The presence of such genes in the QTL region, whose roles in various seed functions are well established, strongly supports the association of the identified QTLs to seed related traits thereby validating the accuracy of the identified QTLs. However, further resolution of the QTLs is required to increase the precision of candidate gene prediction.

## Materials and Methods

### Mapping population and DNA extraction

A segregating population of 177 RILs derived from an intra-specific cross between *C. arietinum* SBD377 (Desi bold seeded, 100 seed weight- 48 g, seed no./plant- 31) and *C. arietinum* BGD112 (Desi small seeded, 100 seed weight-15.4 g, seed no./plant- 153) was grown in the fields at NIPGR, India. Fresh, young leaves were collected and DNA was extracted from 100 mg leaf tissue using the GenElute Plant Genomic DNA Miniprep Kit (Sigma Aldrich, USA) following the manufacturer’s protocol. DNA was quantified using Thermo Scientific Nanodrop 8000 spectrophotometer instrument (Fisher Scientific), and used for GBS library preparation for SNP discovery.

### GBS library preparation, sequencing and SNP genotyping

Genomic DNA from the parents (SBD377 and BGD112) and 93 RILs were used to prepare the libraries for GBS analysis (NxGenBio, India). Libraries were prepared by restriction digestion of DNA of each of the RILs as well as the parents with ApeKI, followed by ligation with barcoded adapters. Ninety six different barcode sequences (ranging in length between 4–10 nucleotides) were used for tagging the samples (Table S10). Appropriate amount of adapters was determined and used for library preparation according to the GBS protocol as described in Elshire *et al.*^14^.

The libraries were pooled and sequenced using Illumina TrueSeq Version 3.0 single end sequencing chemistry with read lengths of 100 bp on HiSeq 2000 Platform. Ninety five samples (plus a blank negative control) were sequenced in one lane. Sequence reads from raw data FASTQ file were processed for sequence filtering through GBS analysis pipeline implemented in TASSEL v3.0^24^,^66^. Briefly, sequences from raw data were filtered by checking for the perfectly matched barcode with the expected four base remnant of the enzyme cut site. Reads with minimum Qscore of 10 across the first 72 bases were considered for further analysis. These reads were sorted and de-multiplexed according to their barcode and further trimmed to 64 bases. The filtered sequence tags were aligned to the already available draft genome sequence of the *desi* chickpea *C. arietinum* ICC4958^2^ using Burrows-Wheeler alignment tool (BWA). Further, pipeline parameters were used for SNP calling and genotyping. To impute the missing values, imputation was carried out using Beagle v3.3.2^44^ with default parameters and ten iterations of the marker sample. Further, the distribution of identified SNPs (raw and filtered) across the 8 chickpea LGs and their frequency (per 100 kb) was estimated and visualized using Circos v0.61^67^.

### Analysis of SNPs for structural and functional relevance

The structural and functional relevance of SNPs was predicted according to the *desi* chickpea genome annotation^25^. Annotation information of *desi* chickpea in GFF format was used to assess the SNP distribution in various genomic regions: intergenic, genic, intragenic and UTRs. To deduce the functional relevance of the SNPs, genes containing SNPs in their exons were functionally classified according to the three principal Gene Ontology (GO) categories using in-house custom perl scripts. The Plant Transcription Factor Database^68^ was used to retrieve peptide sequences of different TFs of five legumes *(C. arietinum, Glycine max, Cajanus cajan, Lotus japonicas and Medicago. truncatula)*. These sequences were utilized to build HMM profiles for all TF families and used to classify genes that contained SNPs in their exons under different TF families.

### Comparative mapping between chickpea and other legumes

The flanking 50 bases of all GBS derived SNPs were physically mapped on the *desi* and *kabuli* chickpea genome assemblies^1^,^2^ using BLASTN. Physical positions of the SNP markers were used for comparative analysis with genome sequences of *G. max* (Glyma1) and *M. truncatula* (v3.5) downloaded from phytozome (v9.1). A comparison between *desi*^25^ and *kabuli*^69^ chickpea genomes was also carried out using physically mapped SNP markers. For comparative study with *Glycine* and *Medicago*, hits matching with minimum of 80% sequence similarity and ≥40% query coverage were retained. However, for comparing genomes of *desi* and *kabuli* chickpeas, only the first hit from BLAST result was retained. Blast hits to scaffolds or regions not anchored to the chromosomal assembly in the target genomes were discarded. The synteny was visualized using Circos software (v0.61)^67^.

### Phenotypic evaluation

The SBD377 X BGD112 RIL population of 177 individuals and parents were grown and evaluated in the fields of Indian Agriculture Research Institute, New Delhi (28.0800° N, 77.1200 E) for mapping. The RILs and their parents were planted in augmented block design with two repetitions. Each experimental block was a single 2 m row plot spaced 0.5 meter between the rows. Preparation of land and pest control was carried out by standard agronomic practices to ensure appropriate plant growth and development. Phenotypic data for the following four traits was evaluated: seed weight (SW), number of seeds per plant (SN), pods per plant (PP) and seeds per pod (SP) for two consecutive years, 2012 and 2013. Average number of seeds per pod was estimated by calculating the number of seeds in five pods and dividing it by 5. Statistical analysis including Pearson’s correlation coefficient among the traits was conducted using XLSTAT^70^.

### Genetic linkage map construction

The allele calls for all genotypes were used for constructing the linkage map using JOINMAP^®^ 4.1 program^71^. Chi square test was performed (p < 0.05) to test the segregation distortion for each marker. Regression mapping method was used with LOD score of 3.5 or higher to group the markers into 8 linkage groups (LGs). Final marker order on each LG was determined by the program RECORD^72^. Distance was calculated using Kosambi’s mapping function^73^ and the intra-specific linkage map was generated with MapChart^74^. The map with best marker order and the least map distance (cM) was considered.

### QTL analysis

For QTL identification the genotyping data of the mapped SNPs on the intra-specific linkage map was integrated with the field phenotyping data of seed weight, seeds per pod, number of seeds per plant and pods per plant. The QTL analysis was performed using QTL Cartographer v2.5^75^ based on composite interval mapping method (CIM) using the forward–backward stepwise regression. For each trait, LOD score threshold was determined by a 1000 permutation test. The software also estimated the percentage of phenotypic variance and additive effect explained by a QTL for a trait. The QTLs were illustrated diagrammatically using Mapchart^74^.

### Identification of candidate genes

To identify putative candidate genes controlling seed and pod traits, sequences underpinning SNP loci flanking QTL intervals were BLAST searched against the reference genome sequence of the *kabuli* chickpea^1^. The corresponding sequences were extracted from the chickpea genome and gene predictions were performed using FGENESH program^76^ to identify the putative candidates. Further, the expression profiles of the candidate genes was analysed by mapping the RNA seq transcriptome data of chickpea tissues such as leaf (SRX048833), root (SRX048832), flower-bud (SRX048834), pod (SRX208035) and developing seed (at four development stages; 10 DAA (Days After Anthesis), 20 DAA, 30 DAA and 40 DAA) (SRX125162). The transcriptome data was retrieved from NCBI and mapped to the putative candidate genes with the help of the 454 Roche gsMapper (Newbler v2.3.5)^77^. The mapped reads were normalized using RPKM measures and these values were used to generate the heatmap using TIGR Multiple Experiment Viewer (MEV) software^78^,^79^.

## Conclusions

The study demonstrates the utility of new tools for facilitating molecular breeding of chickpea. The utility of GBS was revealed for large scale SNP discovery from intra-specific mapping population thereby suggesting its ability to tackle the issue of narrow genetic diversity and low polymorphism detection in chickpea. Additionally, GBS proved to be a useful method for high-throughput genotyping of SNPs, linkage map construction and dissection of QTLs associated with seed traits. Moreover, the integrated approach used here that combined the QTL information with the publicly available genome and transcriptome data helped in identification of putative candidate genes that govern seed traits and would facilitate understanding the molecular mechanisms underlying seed yield in chickpea and pave the way for enhanced chickpea yields.

## Additional Information

**How to cite this article**: Verma, S. *et al.* High-density linkage map construction and mapping of seed trait QTLs in chickpea (*Cicer arietinum* L.) using Genotyping-by-Sequencing (GBS). *Sci. Rep.* **5**, 17512; doi: 10.1038/srep17512 (2015).

## Supplementary Material

Supplementary figures

Supplementary Table S1

Supplementary Table S2

Supplementary Table S3

Supplementary Table S4

Supplementary Table S5

Supplementary Table S6

Supplementary Table S7

Supplementary Table S8

Supplementary Table S9

Supplementary Table S10

## Figures and Tables

**Table 1:** Summary statistics of the chickpea intra-specific genetic linkage map constructed using the RIL population arising out of a cross of (SBD377 X BGD112).

Linkage Group	Total number of mapped markers	Length (cM)	Average interval (cM)
LG1	488	126.97	0.28
LG2	584	136.4	0.23
LG3	543	108.12	0.19
LG4	254	135.84	0.53
LG5	518	116.18	0.22
LG6	373	126.37	0.33
LG7	259	142.42	0.54
LG8	344	114.67	0.33
Total	3363	1006.98	0.33

**Table 2:** Descriptive statistics of measured traits in the F_11_ mapping population of chickpea SBD377 X BGD112.

Trait	SBD377	BGD112	Min.	Max.	Mean	Std. deviation
SN	59	111	9	203	79.09	41.52
100-SW (g)	36.72	15.44	13.35	46.80	24.70	5.92
SP	1.10	1.80	1	2.50	1.63	0.34
PP	100	32	6.50	250	59.18	35.34

**Table 3:** Pearson correlation coefficients for seed traits from the RIL population SBD377 X BGD112.

Variables	SP	SW (g)	SN	PP
SP	**1**			
SW (g)	**−0.268**	**1**		
SN	0.143	**−0.235**	**1**	
PP	0.043	−0.179	**0.757**	**1**

Values in bold are different from 0 with a significance level alpha = 0.05.

**Table 4:** Quantitative trait loci (QTLs) associated with the traits: SP (number of seeds per pod), SW (100-seed weight), SN (number of seeds per plant) and PP (number of pods per plant) and mapped on the intra-specific linkage map of chickpea (SBD377 X BGD112).

QTL	LG	LOD	Marker Interval	Additive effect^a^	% variance
*qSP-1*	1	4.98	CESSR191-CaGSNP1377	−0.15	17.91
*qSP-2*	2	3.30	CaGSNP3957-CaGSNP1136	−0.12	11.09
*qSP-3*	5	3.01	CaGSNP1149-CaGSNP1837	0.12	11.74
*qSP-4*	8	3.34	CaGSNP3914-CaGSNP1491	−0.13	11.25
*qSP-5*	8	3.36	CaGSNP59-CaGSNP209	0.11	10.84
*qSW-1*	1	3.02	CaGSNP2230-PIP152	3.18	10.13
*qSW-2*	2	3.12	CaGSNP260-CaGSNP2024	2.03	12.11
*qSW-3*	5	5.52	CaGSNP65-PIP54	6.23	23.02
*qSW-4*	6	3.66	CaGSNP3407-CaGSNP1133	2.53	14.02
*qSW-5*	7	4.37	CaGSNP1683-CaGSNP2289	3.16	22.31
*qSW-6*	7	3.03	CESSR159-CaGSNP202	−1.89	10.29
*qSW-7*	7	3.03	CaGSNP3029-CaGSNP1100	2.09	10.07
*qSN-1*	4	4.64	CaGSNP1716-CaGSNP164	24.26	18.84
*qSN-2*	6	3.02	CaGSNP1218-CaGSNP628	19.61	9.97
*qSN-3*	7	4.76	CaM0443-CaGSNP3775	−18.97	18.57
*qSN-4*	8	3.17	CaGSNP2706-CaGSNP3777	−17.21	11.34
*qPP-1*	1	3.39	CaGSNP2207-CaGSNP1967	−15.17	12.81
*qPP-2*	2	4.38	CaGSNP693-CaGSNP1374	-16.15	16.93
*qPP-3*	6	3.67	CaGSNP2974-CaGSNP3010	−14.97	14.35
*qPP-4*	8	4.78	CaGSNP2520-CaGSNP1074	49.54	29.71

LG: Linkage Group.

^a^A negative value indicates that given trait is derived from SBD377 and a positive number indicates that the trait is derived from BGD112.
