# Supplementary material for: High-density linkage map construction and mapping of seed trait QTLs in chickpea (Cicer arietinum L.) using Genotyping-by-Sequencing (GBS)
Source: Sci Rep. 2015 Dec 3;5:17512. doi: 10.1038/srep17512 (PMC4668357; doi:10.1038/srep17512)
Supplement: Supplementary figures [file srep17512-s1.pdf]

High-density linkage map construction and mapping of seed trait QTLs in chickpea (*Cicer arietinum* L.) using Genotyping-by-Sequencing (GBS)

Subodh Verma[1], Shefali Gupta[1], Nitesh Bandhiwal[1], Tapan Kumar[2], Chellapilla Bharadwaj[2] and Sabhyata Bhatia[1*]

[1]National Institute of Plant Genome Research, Aruna Asaf Ali Marg, PO Box No. 10531, New Delhi, 110067, India

[2]Indian Agricultural Research Institute, Pusa Campus, New Delhi, 110012, India

[*]To whom correspondence should be addressed

Phone: +91 11 26735159

Fax: +91 11 26741658

Email: sabhyatabhatia@nipgr.ac.in

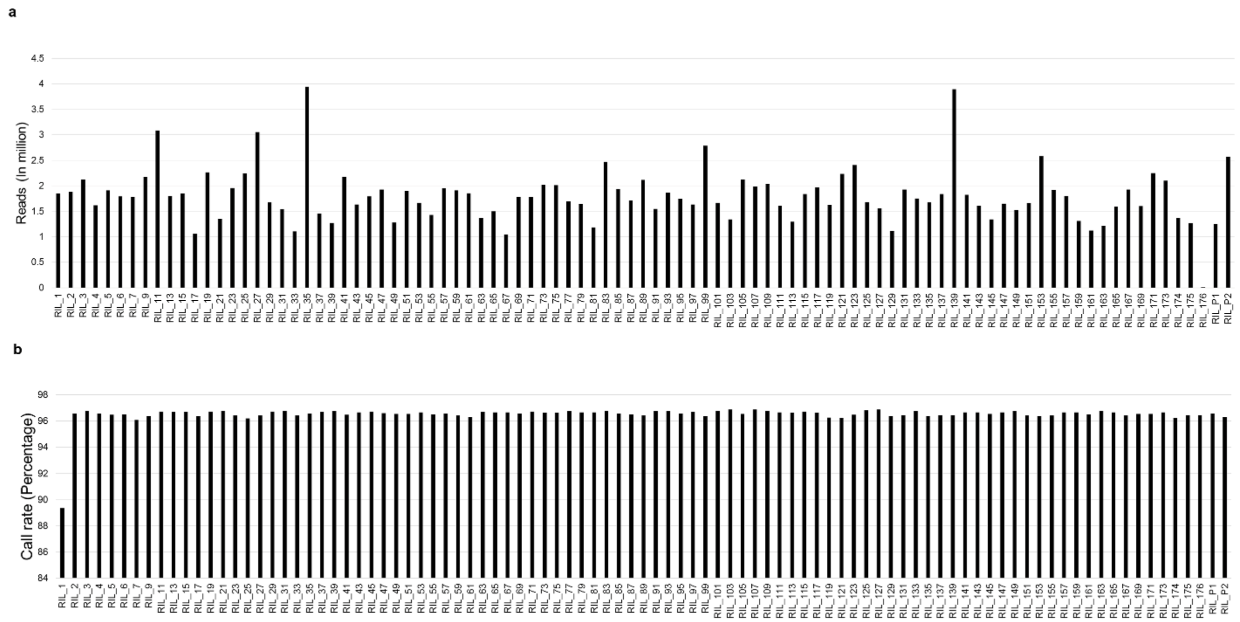

Figure S1. Number of reads in each individual and SNP call rates. **(a)** Number of sequence reads in individual samples. **(b)** Call rates of SNPs (Total No. of positive call/Total SNP for the sample).

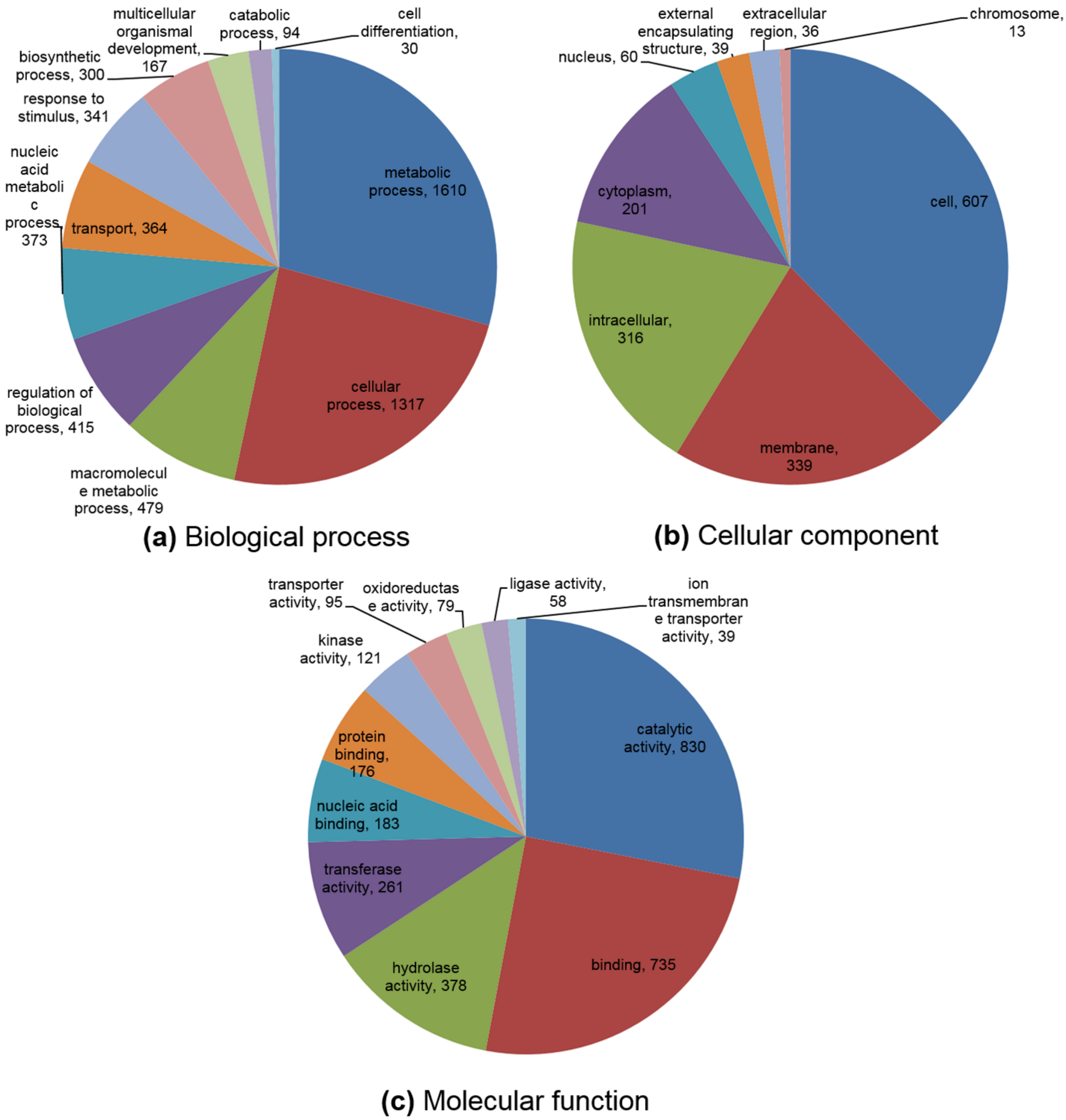

Figure S2. Functional annotation of genes carrying genic SNPs. Genes that contained one or more SNPs in their exons were categorized into functional categories to obtain relationships between the genes function and potential SNPs by Gene Ontology.

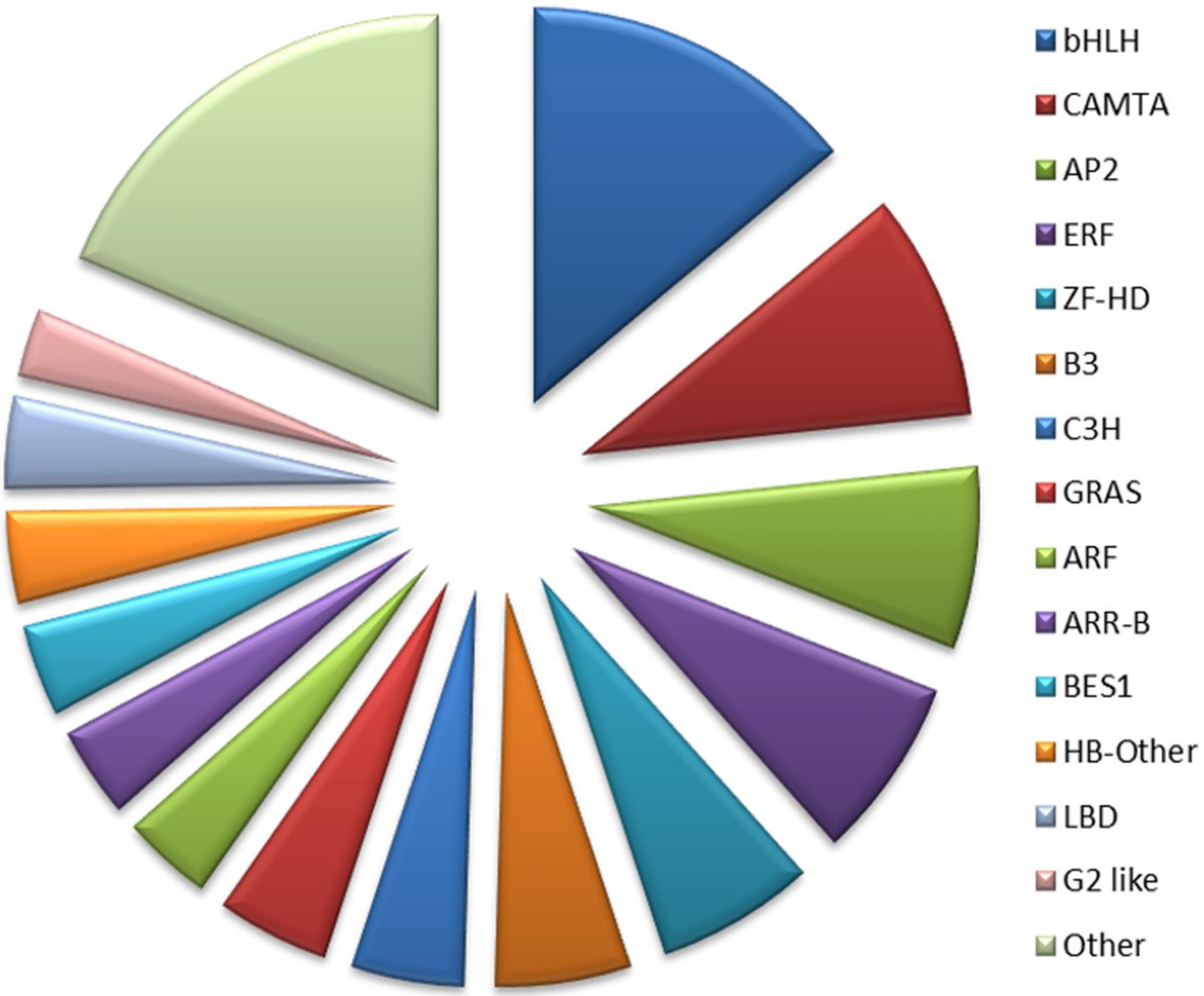

Figure S3. Distribution of genes carrying genic SNPs into transcription factor families.

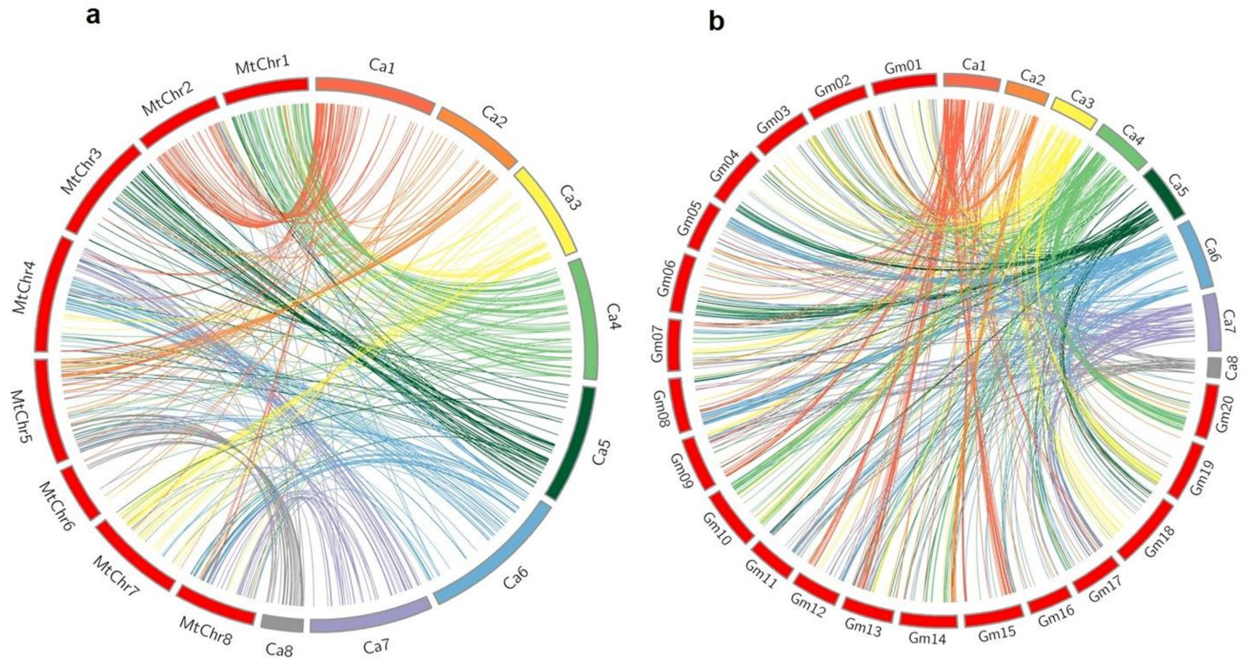

Figure S4. Syntenic relationships of chickpea with closely related legumes. Homologous relationship of *kabuli* chickpea genome with two legume species namely **(a)** *Medicago truncatula* and **(b)** soybean are shown in the Circos diagram. Each line radiating from a chickpea chromosome represents a similarity match found between chickpea and other legumes.

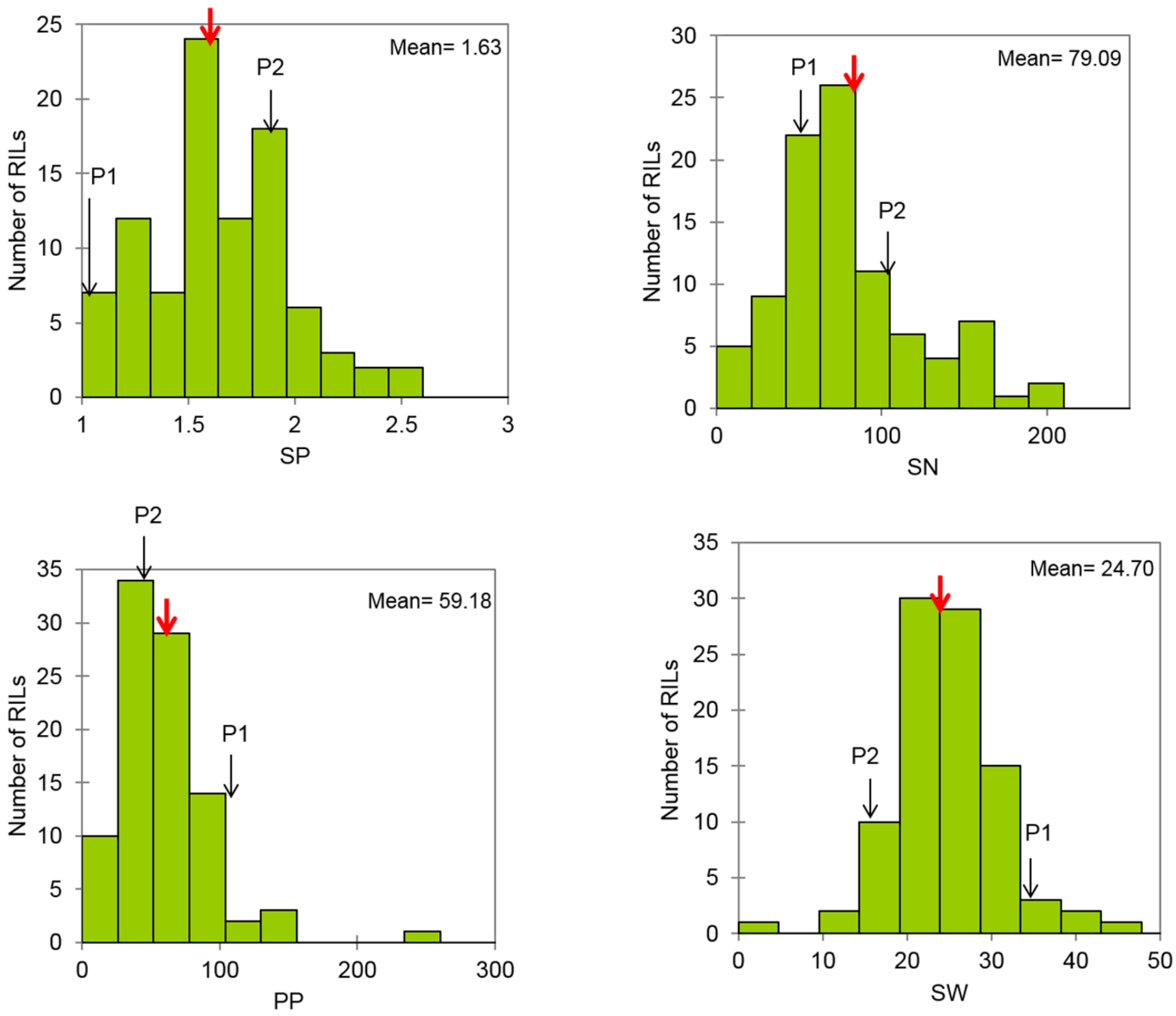

Figure S5. Distribution of the seed weight, seed number/plant, seeds/pod and pods/plant in the F_{11} population derived from the cross SBD377 X BGD112. SW, SP, SN and PP represent seed weight, number of seeds per pod, number of seeds per plant and number of pods per plant respectively. P1 and P2 indicate SBD377 and BGD112, respectively. Red arrows indicate mean values.
